# Targeted Apoptotic Effects of Thymoquinone and Tamoxifen on XIAP Mediated Akt Regulation in Breast Cancer

**DOI:** 10.1371/journal.pone.0061342

**Published:** 2013-04-17

**Authors:** Shashi Rajput, B. N. Prashanth Kumar, Siddik Sarkar, Subhasis Das, Belal Azab, Prasanna K. Santhekadur, Swadesh K. Das, Luni Emdad, Devanand Sarkar, Paul B. Fisher, Mahitosh Mandal

**Affiliations:** 1 School of Medical Science and Technology, Indian Institute of Technology Kharagpur, Kharagpur, West Bengal, India; 2 Department of Human and Molecular Genetics, Virginia Commonwealth Univeristy (VCU) Institute of Molecular Genetics, VCU Massey Cancer Center, Virginia Commonwealth University, School of Medicine, Richmond, Virginia, United States of America; Bauer Research Foundation, United States of America

## Abstract

X-linked inhibitor of apoptosis protein (XIAP) is constitutively expressed endogenous inhibitor of apoptosis, exhibit its antiapoptotic effect by inactivating key caspases such as caspase-3, caspase-7 and caspase-9 and also play pivotal role in rendering cancer chemoresistance. Our studies showed the coadministration of TQ and TAM resulting in a substantial increase in breast cancer cell apoptosis and marked inhibition of cell growth both *in vitro* and *in vivo.* Anti-angiogenic and anti-invasive potential of TQ and TAM was assessed through *in vitro* studies. This novel combinatorial regimen leads to regulation of multiple cell signaling targets including inactivation of Akt and XIAP degradation. At molecular level, TQ and TAM synergistically lowers XIAP expression resulting in binding and activation of caspase-9 in apoptotic cascade, and interfere with cell survival through PI3-K/Akt pathway by inhibiting Akt phosphorylation. Cleaved caspase-9 further processes other intracellular death substrates such as PARP thereby shifting the balance from survival to apoptosis, indicated by rise in the sub-G_1_ cell population. This combination also downregulates the expression of Akt-regulated downstream effectors such as Bcl-xL, Bcl-2 and induce expression of Bax, AIF, cytochrome C and p-27. Consistent with these results, overexpression studies further confirmed the involvement of XIAP and its regulatory action on Akt phosphorylation along with procaspase-9 and PARP cleavage in TQ-TAM coadministrated induced apoptosis. The ability of TQ and TAM in inhibiting XIAP was confirmed through siRNA-XIAP cotransfection studies. This novel modality may be a promising tool in breast cancer treatment.

## Introduction

Tamoxifen (TAM), a nonsteroidal triphenylethylene derivate and selective ER modulator, has been used as a single agent in the treatment of ER-α-positive breast cancer. Clinical response to TAM is associated with both decreased proliferation and increased apoptosis [1], [2]. Studies have revealed that TAM is also effective in treatment of ER-α-negative neoplasia including breast cancer, malignant gliomas, pancreatic carcinomas and melanoma [3], [4]. The apoptotic inducible effect of TAM is not reversible by addition of estrogens, suggesting that ER-α-independent induction of apoptosis could be a central mechanism of action in ER-α-negative breast cancer [5], [6], [7]. In addition, TAM has been shown to cause tumor necrosis and regression via inhibition of angiogenesis in MCF-7 breast tumor xenografts [8].

Extended administration of TAM evokes serious side effects and frequently results in gradual insensitivity to this treatment. Many growth factors, such as epidermal growth factor, insulin-like growth factor and heregulin, confer TAM-insensitivity in ER-α-positive breast cancer cells [9], [10], [11]. Although, the mechanisms by which resistance/insensitivity occur remain unclear. Resistance to TAM is partly mediated through the serine/threonine protein kinase B or Akt (PKB/Akt) in promoting estrogen-independent cell proliferation. The PI3K/Akt pathway plays a crucial role in breast cancer pathogenesis, the up-regulation of which is associated with a more aggressive clinical phenotype and worse clinical outcome for endocrine-treated patients [12], [13]. Previous studies also reported, the dysregulation of anti-apoptotic inhibitor of apoptosis (IAPs) proteins or Bcl-2 proteins, might also contribute to insensitivity to chemotherapy in patients, which are therefore considered as novel therapeutic targets in various cancers. However, we are the first to establish whether antagonists of endogenous anti-apoptotic proteins, such as XIAP, can improve the efficacy of TAM targeted therapies and the possible role in Akt regulation, in breast cancer.

Targeting multiple signaling molecules is essential to induce enhanced apoptosis rather than single molecular target in cancer therapy. Therefore in this study, we employed combination therapy to aim multiple molecular targets in inducing apoptosis, besides reducing the therapeutic dose of TAM [14]. Moreover, combination therapy and the use of naturally occurring innocuous dietary agents achieve greater efficacy in inhibiting tumor cell growth/proliferation and angiogenesis, while at the same time potentially preventing the development of TAM-insensitivity appears to be a viable therapeutic approach [15], [16]. In this view, a dietary phytochemical thymoquinone (TQ) the main active ingredient of the volatile oil of black seed (*Nigella sativa*), employed to overcome TAM evinced serious side effects and assist in inhibiting insensitivity on prolong TAM administration. TQ has been shown to be safe when administered to a wide variety of normal cells including normal mouse kidney cells [17], non-malignant fibroblasts and normal human lung fibroblasts [18], [19], [20], [21], [22]. Previous studies have shown that TQ exhibits inhibitory effects on cell proliferation of many cancer cell types including breast cancer cells [17], [23]. TQ induces cell death, retards human umbilical vein endothelial cell migration and inhibits tumor growth by suppressing NF-κB, Akt activation, and extracellular signal-regulated kinase signaling pathways, as well as angiogenesis [24], [25], [26], [27].

The objective of the current study was to evaluate the potency of TQ in combination with TAM in inhibiting tumor cell growth/proliferation, angiogenesis and to reveal underlying molecular mechanisms involved in apoptosis, while at the same time potentially preventing the development of TAM-insensitivity. TQ treatment synergizes with a low dose of TAM to induce cancer cell death. The synergistic combination exhibited its inhibitory effect through XIAP mediated Akt downregulation.

## Materials and Methods

### Cell Lines

Human breast cancer cell lines MCF-7, MDA-MB-231, MDA-MB-468, T-47D, NIH/3T3 and HaCaT were obtained from the National Center for Cell Science (NCCS), Pune, India and cultured. Normal early passage primary human mammary epithelial cells (HMEC) were obtained from Lonza Clonetics, San Diego and cultured. Cells were incubated at 37°C in a 5% CO_2_ and 95% humidified incubator. Mycoplasma status of all cell lines has been detected through DAPI staining procedures (Data shown for MCF-7 and MDA-MB-231 cells).

### Reagents

Stock solutions of 10 mM TQ and 10 mM TAM (Sigma Aldrich St. Louis, MO, USA), were dissolved in DMSO (Sigma Aldrich St. Louis, MO, USA), stored at −20°C, and diluted in fresh medium just before use. For Western blot analysis, the following antibodies were used: rabbit monoclonal anti-PARP, anti-XIAP, anti-AIF, anti-p-MAPK (Thr202/Tyr204), anti-MAPK, anti-p-Akt (Ser473) and anti-Akt, anti-p-GSK-3β (Ser9), anti-GSK-3β, anti-p-Bad (Ser136), anti-Bad and monoclonal mouse anti-CD-31(Cell Signaling Technology, Beverly, MA, USA) and mouse anti-Ki-67 (Santa Cruz Biotechnology, Santa Cruz, CA, USA), mouse monoclonal anti-caspase-9 (BD Pharmingen, San Jose, CA, USA), mouse monoclonal anti-β-actin (Sigma Aldrich, St. Louis, MO, USA), mouse monoclonal anti-Bcl-2, anti-Bax, anti-p53, anti-Bcl-xl, horseradish peroxidase-conjugated goat anti-rabbit IgG and goat anti-mouse IgG (Santa Cruz Biotechnology, Santa Cruz, CA, USA). Antibodies dilutions have been made as per the manufacturer’s instruction for primary and secondary antibodies were 1∶1000 and 1∶2000, respectively. The pcDNA3-XIAP-Myc plasmid (Addgene plasmid 11833) was kindly provided by Dr. Guy Salvesen (University of California, San Diego, CA, USA) pcDNA3.1(-) was purchased from Invitrogen and siRNA XIAP was purchased from Cell Signaling Technology, Beverly, MA, USA. FuGENE® HD Transfection Reagent (Roche Applied Science, Mannheim, Germany), Opti-MEM® I Reduced Serum Media, Fetal bovine serum (FBS) (Gibco-BRL, Invitrogen Corporation, CA, USA), Bovine serum albumin (BSA), Trypsin (Himedia, Mumbai, INDIA) Chemiluminescent peroxidase substrate, Propidium iodide (PI), 4′, 6-diamidino-2-phenylindole (DAPI) and 3-(4, 5-dimethylthiazol-2-yl)-2, 5-diphenyltetrazolium bromide (MTT) (Sigma-Aldrich, St. Louis, MO, USA) were purchased from the corresponding company. Stock solutions of PI, DAPI and MTT were prepared by dissolving 1 mg of each compound in 1 ml PBS. The solution was protected from light, stored at 4°C, and used within 1 month. Stock concentrations of 10 mg/ml RNase A (Sigma-Aldrich, St. Louis, MO, USA) were prepared and kept at −20°C.

### Evaluation of TQ and/or TAM Cytotoxicity

Cells were harvested in the logarithmic phase of growth; cell suspensions were dispensed (200 µl) into 96-well tissue culture plates at an optimized concentration of 1×10^4^ cells/well in complete medium. After 24 h, cells were treated in quadruplicate with TQ (0.01–60 µM) and/or TAM (0.01–40 µM), or with DMSO (0.01%) control treatment, and incubated for 48 h. Cell viability was measured by MTT dye reduction assay at 540 nm [28] with slight modifications in protocol. The dose-effect curves were analyzed using Prism software (GraphPad Prism, CA, USA).

### Flow Cytometric Analysis

To study the combination effect of TQ and TAM, MCF-7 and MDA-MB-231 Cells were treated with respective IC_50_ values for 48 h after seeding in 60-mm tissue culture plates. After treatment, cells were collected, washed and incubated in 70% ethanol, kept at −20°C overnight for fixation. Cells were centrifuged, washed and then incubated with PI solution (40 µg/ml PI, 100 µg/ml RNase A in PBS) at 37°C for 1 h. The distribution of cells in the different cell-cycle phases was analyzed from the DNA histogram using Becton-Dickinson FACS Calibur and Cell Quest software, CA, USA.

The procedure of Annexin V-FITC with propidium iodide (PI) staining was carried out according to the manufacturer's protocol (Sigma-Aldrich, St. Louis, MO, USA). After TQ and/or TAM treatment for 24 h, cells were trypsinized, washed with binding buffer, and resuspended in Annexin V-FITC and PI added to the binding buffer for 15 min under dark conditions [29], [30]. The cell samples were analyzed immediately by flow cytometry.

### Western Blotting Analysis

MCF-7 and MDA-MB-231 cells were treated with TQ and/or TAM at their respective IC_50_ dose for 24 h. For phosphoprotein studies, experimental wells were treated with TQ and/or TAM at respective IC_50_ concentrations, whereas the control wells were treated with 0.01% DMSO for 1 h. Then, cells were activated with recombinant human EGF (25 ng/ml) for 30 min. The cells were then scraped and lysed in Nonidet P-40 lysis buffer. Cell extracts (50 µg protein) were separated on sodium dodecyl sulfate-polyacrylamide electrophoretic gel (SDS-PAGE) and transferred to nitrocellulose membranes, which were blocked in 3% BSA for 2 h. After blocking, the membranes were incubated with primary antibodies overnight at 4°C and then with horseradish peroxidase–conjugated secondary antibody for 2 h at room temperature. Proteins were visualized using the chemiluminescence substrate and exposed to Kodak X-OMAT AR autoradiography film (Eastman Kodak, Rochester, NY, USA). Cytosolic protein extracts were isolated based on previous reported method [31].

### Transfection Studies

Breast cancer cells were plated in 70-mm petri dishes at a density over 4×10^5^ per plate in DMEM media supplemented with 10% FBS. After growth for 16 to 20 h, cells were starved for 6 h with 2% FBS. 70–80% confluent cells were transiently transfected with 5 µg of pcDNA3-XIAP-Myc with 7.5 µl of FuGENE® HD Transfection Reagent in 100 µl of Opti-MEM® I Reduced Serum Media (Gibco-BRL, Invitrogen Corporation, CA, USA) according to manufacturer’s protocol (Roche Diagnostics GmbH, Mannheim, Germany). After 24 h transfection, the mix was replaced with complete media containing no compound or TQ and/or TAM for 24 h. The cells were then scraped and pelleted, and the protein expression profiles were determined by western blotting following similar protocol as mentioned above. Densitometric analysis of blots was performed by ImageMaster 2D Platinum 7.0 Software (GE Healthcare Life Sciences, NJ, USA).

In cotransfection studies, using FuGENE® HD Transfection Reagent, MCF-7 and MDA-MB-231 cells were transfected/co-transfected with 2 µl of 300 nM siRNA-XIAP and 5 µg of pcDNA3-XIAP-Myc. The transfective reactives for each transfection were prepared using 2 µl of 300 nM siRNA-XIAP, 5 µg of pcDNA3-XIAP-Myc and 7.5 µl of FuGENE® HD Transfection Reagent in 100 µl of Opti-MEM® I Reduced Serum Media and the tubes were left at room temperature for 45 min. After incubation, transfection procedures were carried out according to manufacturer’s protocol (Roche Diagnostics GmbH, Mannheim, Germany).

### *In vivo* Xenograft Studies

Tumor response to TQ and/or TAM was studied using a human breast cancer nude mouse xenograft model. Our study was approved by the Department of Biotechnology (DBT), INDIA under the project number: E-1/MMSMST/12, at Indian Institute of Technology Kharagpur, INDIA and the mice were maintained in accordance with the institute animal ethical committee (IAEC) guidelines approved by Indian Council of Medical Research (ICMR), New Delhi. Mice were housed and acclimatized in pathogen free environment at institute animal facility for 1 week prior to injection with MDA-MB-231 cells. Exponentially growing MDA-MB-231 cells were harvested and a tumorigenic dose of 2.5×10^6^ cells in Matrigel (0.5 mg/ml) were injected subcutaneously (s.c.) in 6–7 week-old female athymic BALB/c (nu+/nu+) mice [32], [33]. Tumors were allowed to grow for 7 d; all of the mice were then weighed, and all of the tumors were measured using microcalipers. Tumor volume was calculated using the formula (A) (B^2^) π/6, where A was the length of the longest aspect of the tumor, and B was the length of the tumor perpendicular to A. All mice were randomized into four groups, containing 5 mice per group. Group one, the control group received 1% poysorbate resuspended in deionized water, the second group was treated with TQ (20 mg/kg body weight) in alternative days s.c. for 3 weeks, group three received TAM 5 mg/kg/d orally 2 times a week for 3 weeks and group four was treated together with TQ 20 mg/kg (s.c.) in alternative days and TAM 5 mg/kg/d orally 2 times a week for 3 weeks. The doses were selected based on previous experiments. Tumors were measured at the end of every week. After 4 weeks of treatment, mice were euthanized, and the tumors were measured again. During the experiment, mice were examined twice weekly for weight loss.

### Immunohistochemical Analysis (IHC)

Immunohistochemistry was performed with the following antibodies: rabbit anti-XIAP, anti-p-Akt (Ser473) and anti-Akt, anti-p-GSK-3β (Ser9), anti-GSK-3β, anti-p-Bad (Ser136), anti-Bad and monoclonal mouse anti-CD31 (Cell Signaling Technology, Beverly, MA, USA) and mouse anti-Ki-67. IHC studies were performed as described previously with slight modifications [32]. Images were captured at magnification 10× and digitized using FLUOVIEW 1000 (Version 1.2.4.0) imaging software (TYO, Japan).

### Statistical Analysis

All the statistical analysis was performed by Graphpad Prism 5 software. Data are presented using mean ± S.D. The statistical significance was determined by using one-way analysis of variance (ANOVA). ***P<0.001 and **P<0.05 were considered significant.

## Results

### TQ Aggrandize TAM’s Ability in Disrupting Cell Viability of Breast Cancer Cells

**Figure 1:**
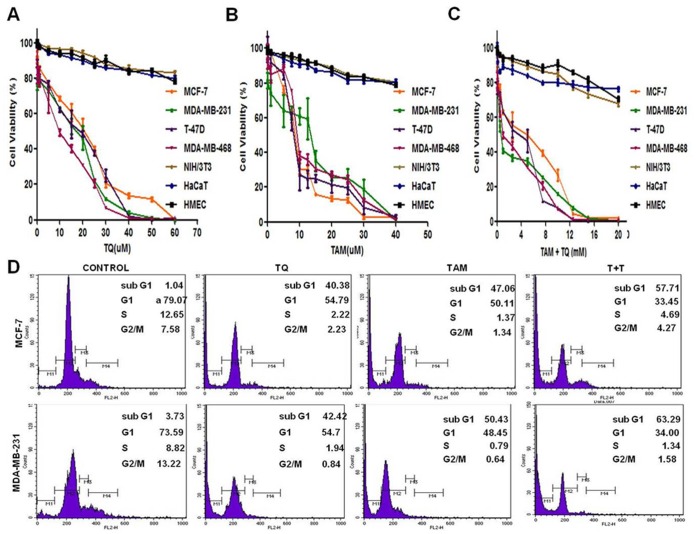
Dose-dependent growth inhibition of breast cancer cells by thymoquinone (TQ) and/or tamoxifen (TAM). Cell viability assays of MCF-7, MDA-MB-231, T-47D, and MDA-MB-468, NIH/3T3, HaCaT and HMEC cells treated with: (A) TQ, (B) TAM and (C) TQ-TAM (varying concentrations of TAM along with 5 µM TQ for 48 h). Points represent mean ± S.E. (n = 4), p<0.05. (D) Representative histogram plot of MCF-7 and MDA-MB-231 breast cancer cells showing distribution in the different phases of the cell cycle after 48 h treatment, determined by ﬂow cytometry after staining cells with PI. Each individual experiment has been repeated three times.

To determine the effect of TQ and TAM alone and in combination on the cell viability of breast cancer cells *in vitro*, ER-α-positive MCF-7 and T-47D cells and ER-α-negative MDA-MB-231 and MDA-MB-468 cells were initially treated with increasing concentrations of either TQ (0.01 µM–60 µM) or TAM (0.01 µM–40 µM) (Fig. 1A and 1B). Combination treatments were carried out by varying TAM (0.01 µM–20 µM) in the presence of 5 µM TQ. TQ treatment on ER-α-positive MCF-7 and T-47D cells resulted in an IC_50_ of 19.78±0.04 µM and 18.06±0.71 µM, respectively, whereas ER-α-negative MDA-MB-231 and MDA-MB-468 cells had an IC_50_ of 15.55±0.68 µM and 12.30±0.62 µM, respectively. TAM treatment of ER-α-positive MCF-7 and T-47D cells produced an IC_50_ of 9.06±0.29 µM and 8.99±0.55 µM, respectively, whereas ER-α-negative MDA-MB-231 and MDA-MB-468 cells had an IC_50_ of 13.05±0.91 µM and 11.56±0.65 µM, respectively. Combined TQ (5 µM) treatment with varying concentrations of TAM resulted in a leftward shift of the concentration-response curve (Fig. 1C). The IC_50_ values of the four cell lines MCF-7, T-47D, MDA-MB-231 and MDA-MB-468 were 5.09±0.35 µM, 3.51±0.26 µM, 2.94±0.47 µM and 2.80±0.29 µM, respectively, indicating enhanced cytotoxicity. TQ (0.01–25 µM) and TAM (0.01–15 µM) and in combination TQ (5 µM)+TAM (0.01–7.5 µM) showed negligible/less toxicity in NIH/3T3, HaCaT and HMEC normal cell lines.

### TQ Enhances TAM Induced Apoptosis and Growth Inhibition

The effects of TQ and/or TAM on MCF-7 and MDA-MB-231 cell cycle were analyzed (Fig. 1D). TQ or TAM treated MCF-7 (IC_50_ of TQ 20 µM and TAM 9 µM) and MDA-MB-231 (IC_50_ of TQ 16 µM and TAM 13 µM) cells had an increased percentage of apoptotic cells (sub-G_1_ phase) compared to untreated controls (0.27±1.6%). Interestingly, a low dose combination of TQ and TAM (TQ 5 µM+TAM 5 µM for MCF-7 and TQ 5 µM and TAM 3 µM for MDA-MB-231) had a significantly higher percentage of apoptotic cells than higher doses of either drug alone. Cell apoptosis studies were further assessed through morphological and nuclear changes (Live/dead assay, Trypan blue and DAPI staining) (methods, results and figures are provided in Data S1, Fig. S1).

### TQ plus TAM Inhibit *in vivo* Angiogenesis and *in vitro* Tube-like Capillary Formation

**Figure 2:**
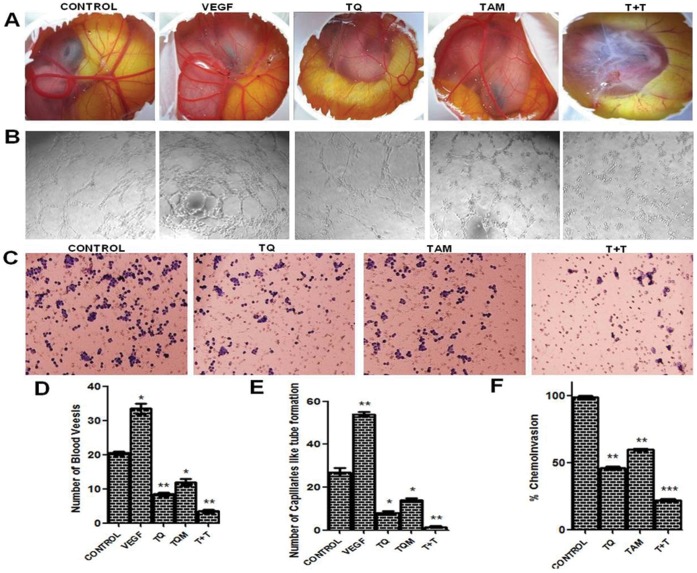
Anti-angiogenic and anti-neovascularization potential of TQ and/or TAM monitored in angiogenic models. (A) Chorioallantoic membrane (CAM) assays. CAMs were implanted with sponges loaded with: serum-free medium (SFM) alone as control; SFM supplemented with vascular endothelial growth factor (VEGF) alone; and with TQ and/or TAM. Combination treatment inhibited the VEGF-induced angiogenic response. (B) Inhibition of capillary tube formation *in vitro* (HUVECs assay). HUVECs were seeded (7.5×10^3^ cells/well) into a 96-well tissue culture plate coated with 50 µl Matrigel. Then, TQ and/or TAM were added. Cells were incubated in HUVEC growth medium in a 37°C, 5% CO_2_ incubator. Tube formation was observed for 24 h and images were taken (magnification of 10×). (C) Representative photomicrographs of Boyden chamber assays of cell invasion through Matrigel. (D) Data are presented as means ± S.D. of number of blood vessel formation in the CAM assay. P<0.05. (E) Number of capillary-like structures was measured in HUVEC capillary formation assay by light microscopy after 24 h in four independent experiments. (F) Data represent the average percentage of cells (± S.D.) invading the Boyden chamber inserts. Data are presented as means ± S.D. P<0.05. Each individual experiment has been repeated three times.

The CAM model was used to investigate the effect of TQ and/or TAM on angiogenesis *in vivo* [34] (Materials and method section was included in Data S1) [35], [36]. As shown in Fig. 2A and 2D, the chorioallantoic membranes in the serum-free medium (SFM) alone as control group do not show any observable avascular zone around the implanted filter paper. However, TQ and/or TAM inhibited the development of new embryonic capillaries and produced an avascular zone around the implanted filter papers. The inhibition of angiogenesis was more prominent following TQ-TAM combination treatment than either drug alone. However, no apparent toxicity was observed in the embryos used in this experiment. Next, we performed tube formation assays in HUVEC cells, which are widely used as *in vitro* assays for angiogenesis. After 24 h, HUVECs treated with serum-free medium rapidly aligned and formed hollow, tube-like structures (Fig. 2B and 2E). In contrast, HUVECs treated with TQ and TAM in combination showed a significant reduction of tube formation in comparison to TQ or TAM alone. Collectively, these results suggest that TQ enhances the anti-angiogenic action of TAM by inhibiting HUVEC differentiation into tube-like structures during angiogenesis.

### TQ Augments TAM’s Efficacy in Inhibiting *in vitro* Cell Migration and Invasion

To determine the effect of TQ and/or TAM on migration, *in vitro* wound (scratch) assays were performed in both MCF-7 and MDA-MB-231 cells (methods, results and figures are provided in Data S1, Fig. S2). To examine the effect of TQ and/or TAM on the invasive ability of MDA-MB-231 cells, Boyden chambers coated with Matrigel were used (Materials and methods section was included in Data S1) [37], [38]. MDA-MB-231 cells treated with TQ and/or TAM for 24 h were plated in the upper chamber, and the number of cells that moved to the underside of the coated membrane was counted 12 h later using a light microscope. The chambers were stained with hematoxylin- and eosin (H&E) staining and analyzed by photography. These experiments demonstrated that the number of cells that invaded the lower chamber was significantly decreased after 24 h following combination treatment in comparison to either drug alone (Fig. 2C and 2F).

### Synergistic Action of TQ and TAM Alters Expression Profiles of Cell Cycle Regulatory and Apoptotic Proteins

**Figure 3:**
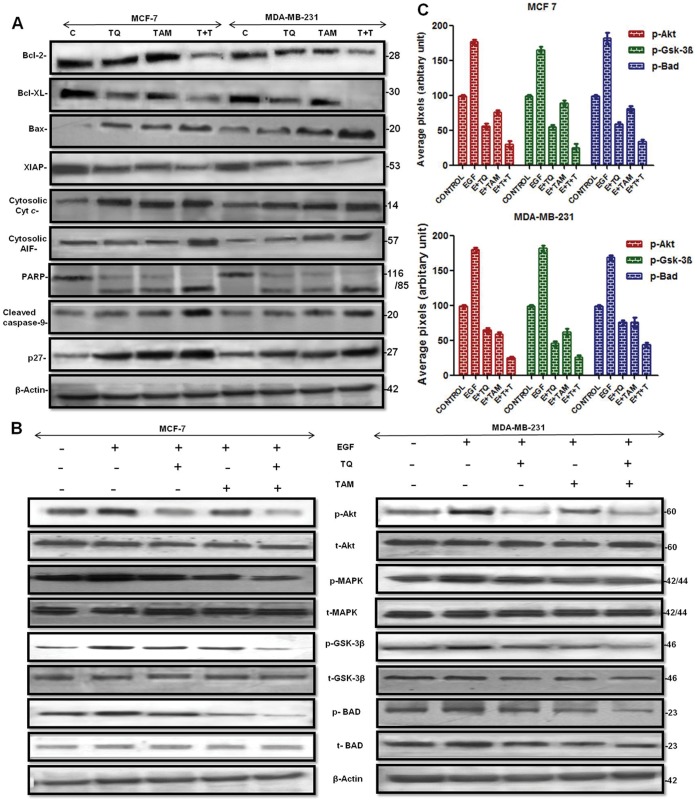
Phosphoprotein and total protein expression profiles of MCF-7 and MDA-MB-231 breast cancer cells treated with TQ and/or TAM. (A) Pro- and anti-apoptotic protein expression profiles by western blotting assays. (B) Autophosphorylation of p-MAPK (Thr42/22), p-Akt (Ser473), p-Bad (Ser136) and p-Gsk-3-β (Ser9) was evaluated along with the total protein expression *in vitro* in cells grown in serum-free medium and cells stimulated with recombinant human EGF (25 ng/ml) for 30 min alone and in the presence of drug(s). β-actin protein expression was used as an internal probe for equal protein loading. (C) Densitometric analysis of phospho protein levels in western blot where each bar represents three independent experiments; P<0.05 (t-test).

Western blotting confirmed the antiproliferative and apoptotic role of TQ in modulating TAM’s ability to decrease cell viability. There was a decrease in the levels of anti-apoptotic Bcl-2 and Bcl-xL expression, and an increase in the levels of pro-apoptotic Bax, p27, cytosolic AIF and cytochrome C proteins following combination treatment (Fig. 3A). Release of cytochrome C from mitochondria leads to Caspase-9 activation (cysteine-aspartate proteases) and the downstream caspase cascade by cleaving at specific sites and hetero-dimerizing to produce the active caspases that play a central role in the execution-phase of cell apoptosis (Fig. 3A). Apoptotic cell death induced by activation of caspase-9 is also regulated by intracellular XIAP protein levels, which were reduced to a greater extent following TQ-TAM combination treatment than with either drug alone. The activated caspase-9 (37-Kd) processes other caspases, including various death substrates such as poly (ADP-ribose) polymerase (PARP) and other molecules, at conserved aspartic acid residues. Cleavage of PARP was observed in MCF-7 and MDA-MB-231 cells treated with either TQ or TAM as compared to control. The cleavage was more profound following combination treatment as there was increased expression of the 85-Kd fragment (cleaved PARP) with almost absence of the 116-Kd fragment (uncleaved PARP) (Fig. 3A).

### TQ-TAM Interfere with Akt Signaling through p-Bad and p-GSK-3β Down Regulation

Because the PI3K/Akt signaling pathway is involved in cell survival signaling and insensitivity in human breast cancer, [39] we determined the potential attenuation of this pathway by TQ-TAM treatment. Western blotting analysis (Fig. 3B and 3C) confirmed a specific reduction in the p-Akt protein in MCF-7 and MDA-MB-231 cells treated with TQ-TAM, as compared with that of untreated controls as well as cells undergoing individual drug treatment. The total Akt level, however remained unaffected by all treatment conditions. The expression levels of Akt downstream substrates including Bad and GSK-3β were assessed (Fig. 3B and 3C). The combination treatment also decreased p-Bad, p-MAPK and p-GSK-3β without affecting their non-phosphorylated forms. Taken together, these data suggest that regulation of the Akt pathway is intimately associated with TQ-TAM-induced growth inhibition and apoptosis.

### XIAP Overexpression Induce Akt Phosphorylation and Effects its Downstream Targets

**Figure 4:**
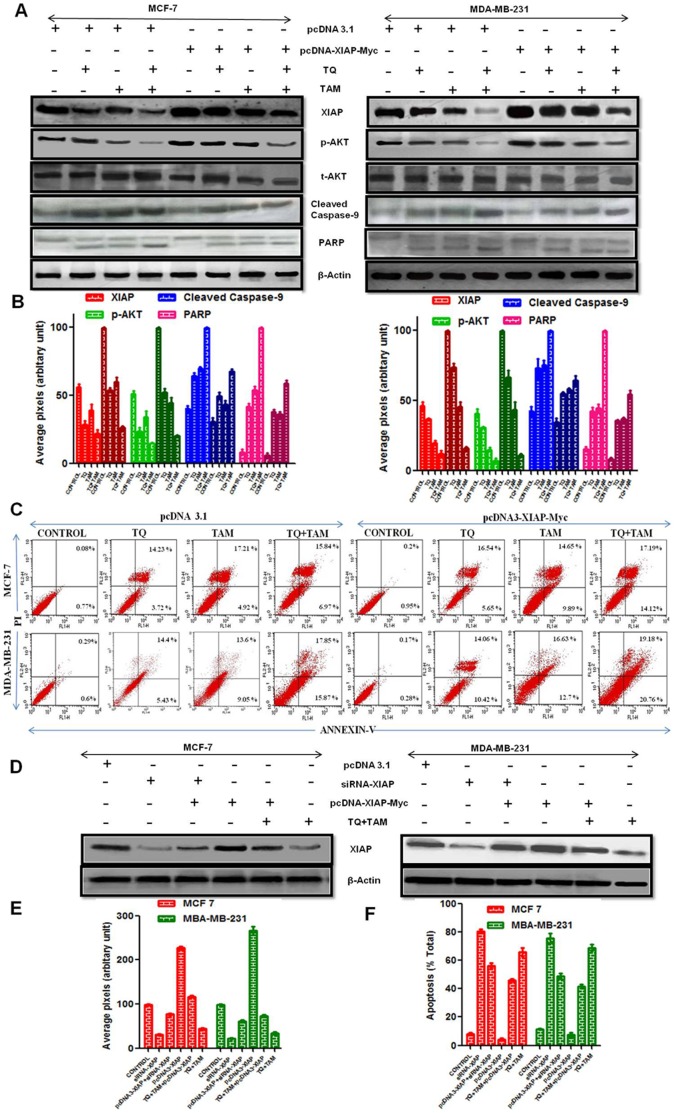
Overexpression of XIAP inhibits TQ and TAM-induced apoptosis. (A) MCF-7 and MDA-MB-231 cells were transfected with pcDNA3-XIAP-Myc construct. Cell lysates were subjected to western blotting analysis using XIAP, p-Akt, caspase-9, PARP, and β-Actin (Equal loading marker) antibodies. (B) Densitometric analysis of XIAP, p-Akt, caspase-9 and PARP protein levels in transfection blot where each bar represents three independent experiments; P<0.05 (t-test). Light and dark color bars represent pcDNA3.1 and pcDNA3-XIAP-Myc transfected cells, respectively. (C) Early and late apoptosis of pcDNA-XIAP-Myc cotransfected cells were analysed through flow cytometer after staining the cells with Annexin V-FITC and PI. (D) MCF-7 and MDA-MB-231 cells were cotransfected with siRNA-XIAP and pcDNA3-XIAP-Myc construct. Cell lysates were subjected to western blotting analysis using XIAP. (E) Densitometric analysis of XIAP protein levels in cotransfection blot where each bar represents three independent experiments; P<0.05 (t-test). (F) At the end of the treatment period, cells were collected from annexin studies (for apoptotic cell count).

To confirm the involvement of XIAP in TQ- and/or TAM-mediated apoptotic cell death, we overexpressed XIAP in MCF-7 and MDA-MB-231 cells lines by transfection with pcDNA3-XIAP-Myc (Fig. 4A, 4B and 4C). XIAP is a member of the inhibitor of apoptosis protein family that potentially inhibits apoptotic cell death by specifically targeting caspase-9 [40]. As shown in annexin studies (Fig. 4C), vector control cells underwent apoptosis (∼37% and ∼40% in MCF-7 and MDA-MB-231 cells, respectively) when treated in combination with TQ and TAM for 24 h. By comparison, cells overexpressing XIAP underwent ∼25% and ∼26% apoptosis in MCF-7 and MDA-MB-231 cells, respectively. Western blot analysis of coadministered TQ-TAM revealed enhanced inhibition of p-Akt levels in XIAP overexpressed cells while that of control. In the downstream targets of p-Akt we observed extensive cleavage of caspase-9 and PARP in comparison to pcDNA 3.1-transfected cells (Fig. 4A), which decreased modestly in XIAP overexpressed cells confirming the involvement of XIAP in TQ- and TAM-mediated cell death. Distinguishingly, our finding revealed two and three fold decrease of XIAP protein profile in MCF-7 and MDA-MB-231 cells, respectively in dual drug treated XIAP overexpressed cells in comparison to control.

### Cotransfection Studies Revealed the Efficacy of Synergistic Combination in Inhibiting XIAP Expression in Breast Cancer Cells

Indeed, we checked the potency of our combination in inhibiting XIAP in both MCF-7 and MDA-MB-231 cells with respect to siRNA-XIAP as positive control. In normal cells, the extent of XIAP inhibition by coadministration of TQ-TAM showed analogous inhibition profiles as that of control siRNA-XIAP. The fold decrease of protein expression was around 2.5 and 2 in siRNA-XIAP and dual drug treated, respectively in both the cell lines corresponding to control. pcDNA3-XIAP-Myc transfected cells showed elevated levels of XIAP expression in comparison with normal cells (Fig. 4D and 4E). Besides its overexpression, synergistic combination showed analogous inhibition of XIAP to that of siRNA-XIAP and pcDNA3-XIAP-Myc cotransfected cells with respect to pcDNA3.1 transfected control. Synonymously, overexpressed cells displayed 1.7 and 1.5 fold decrease of XIAP expression in siRNA-XIAP and dual drug treated, respectively in both MCF-7 and MDA-MB-231 cells. The percent apoptosis in cotransfected cells was demonstrated through annexin PI studies (Fig. 4F). Collectively, from the above results synergistic combination inhibited XIAP despite of its overexpression, concluding its potency and precise targeting of XIAP for apoptosis in breast cancer.

### Effective Role of TQ and TAM in Reducing Tumor in MDA-MB-231 Mice Xenografts Models

**Figure 5:**
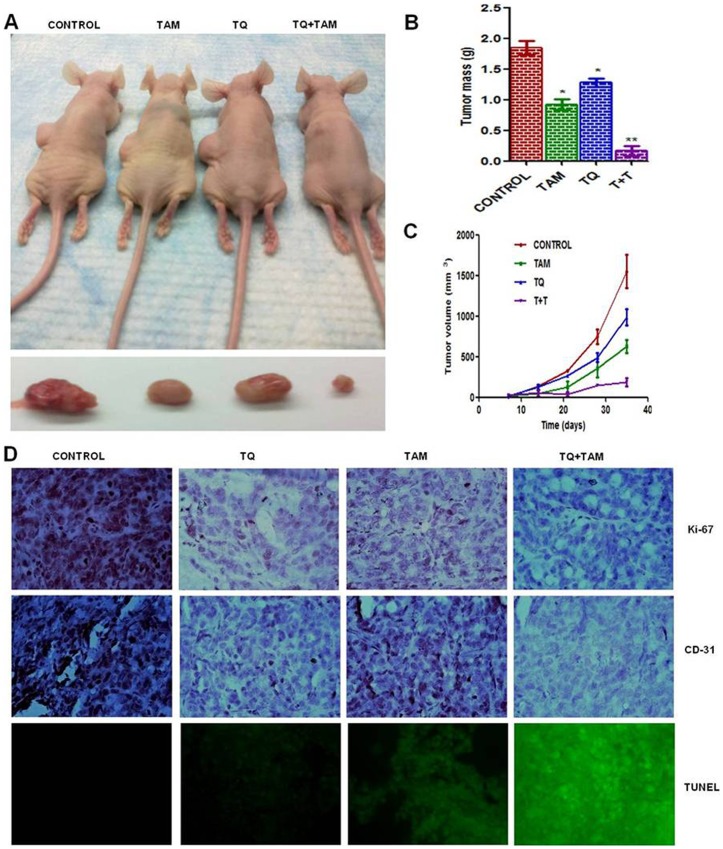
Antitumor activity of TQ and/or TAM in MDA-MB-231 human breast carcinoma xenografts. Nude mice bearing MDA-MB-231 xenografts were treated with TQ (20 mg/kg body weight) s.c. on alternate days and/or TAM 5 mg/kg/day orally 2 times a week, starting 1 week after tumor cell implantation and continued for 3 weeks. Nude mice (A) bearing xenografted MDA-MB-231 breast carcinoma cells along with the images of the excised tumors at the time of sacrifice, (B) Bar graph represents tumor mass in grams, and (C) tumor volume in mm^3^, after 4 weeks of tumor cells implantation provided as mean ± S.D. (n = 5), p<0.05, when compared with the cancer control group. (D) Tumors from different treatment groups underwent immunohistochemical analysis for expression of Ki-67 (cell proliferation marker), CD-31 (angiogenesis marker) and TUNEL (apoptotic marker). Representative pictures were taken at 10× magnification.

We further tested the combination effects of TQ plus TAM on *in vivo* tumor growth, using MDA-MB-231 breast cancer cells in a nude mouse xenograft model (Fig. 5A). There was a significant reduction in tumor size and tumor mass (p = 0.0002, n = 5, unpaired t-test) (Fig. 5B and 5C) after combination drug treatment compared to either drug alone. In the mouse xenograft model, either TQ or TAM given alone inhibited the growth of MDA-MB-231 cells in nude mice 985.135±36.123 mm^3^ in TQ-treated and 629.689±27.646 mm^3^ in TAM-treated, versus 1551.249±56.931 mm^3^ in control animals after 4 weeks of tumor cells implantation (Fig. 5A and 5C). We also observed more profound antitumor effect following TQ-TAM combination treatment than with either agent given alone (187.561±52.480 mm^3^, P<0.0035, ANOVA test). There was no weight difference between the animal groups, and we observed no toxicity. Immunohistochemical studies showed a decrease in Ki-67 positivity and an increase in DNA fragmentation, as shown by antibody staining and TUNEL assays, respectively (Fig. 5D), in TQ-TAM-treated compared to control mice bearing tumors, thereby confirming a role of this combination in exerting antiproliferative and apoptotic-promoting effects. Additionally, there was a decrease in CD-31 staining in MDA-MB-231 xenografts after combination treatment (Fig. 5D), suggesting an inhibitory effect on angiogenesis.

### TQ and/or TAM Block Akt-mediated Signaling in Breast Carcinoma Cells Growing Subcutaneously in Nude Mice

**Figure 6:**
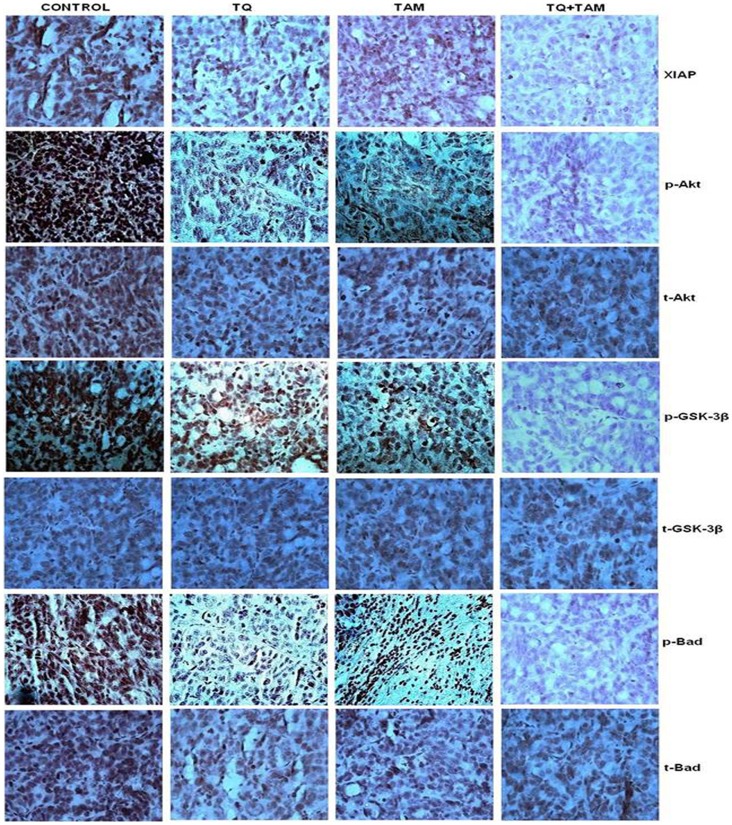
Immunohistochemistry of TQ and/or TAM-treated MDA-MB-231 human breast carcinoma xenografts. Paraffin-embedded sections of MDA-MB-231 bearing tumors in nude mice were processed and IHC was done. IHC of XIAP, p-Akt (Ser473), p-Gsk-3-β (Ser136) and p-Bad (Ser136) (along with total Akt, Gsk-3-β and Bad) representative of three independent experiments. Representative pictures were taken at 10× magnification.

Immunohistochemical studies revealed the reduced levels of XIAP expression in combination treatment group while the expression level of total Akt, GSK-3β and Bad did not vary significantly among tumors from all the four groups (Fig. 6). In contrast, Akt phosphorylation was markedly reduced in tumors treated with TQ-TAM than with either drug alone. When antibodies specific to serine phosphorylated (activated) Akt was used, receptor showed high levels of phosphorylation in the absence of treatment. Levels of Akt phosphorylation were markedly reduced in tumors treated with TQ and/or TAM in comparison with controls. The status of a major downstream target of the Akt pathway, GSK-3β, was also assessed by immunohistochemical analysis. There were only negligible or no changes in the levels of expression of total Akt, GSK-3β and Bad proteins in the treatment vs. control groups. However, the phosphorylation status of GSK-3β and Bad proteins, although high in the control, was distinctly downregulated in the mice treated with TQ and TAM (Fig. 6). Expression studies of Ki-67, CD-31 and TUNEL confirmed the anti-proliferative, anti-angiogenic and anti-apoptotic effects of TQ and TAM (Fig. 5D). These results correlate directly with the findings described for the *in vitro* experiments.

## Discussion

TAM has been extensively used for decades to treat a wide-range of cancers [3]. Despite of its success in breast cancer therapy it also renders undesirable side effects which contributes to poor clinical quality life of patients, thus made in search for benign alternatives [13]. The dose-escalation necessary to overcome even a small increase in cellular resistance can cause severe dose-limiting cytotoxicity to normal tissue. Consequently, strategies using dual agents that act through distinct molecular mechanisms, rather than using single agents, represent a potentially viable alternative for achieving higher cure rates with less toxicity following cancer chemotherapy. Recently, there has been an increasing interest in evaluating synergistic cancer cell cytotoxicity by combining chemotherapeutic agents with highly promising and relatively innocuous dietary phytochemicals [41], [42]. In this regard, we had chosen TQ as an adjuvant to TAM mediated therapy in breast cancer.

XIAP is an eye catching member of the IAP family, indulged in malignant effects of tumor cells especially cell invasion, proliferation, angiogenesis and chemoresistance. However, elevated XIAP expression has been found to be tightly associated with malignancy property, the underlying molecular mechanisms giving rise to XIAP accumulation in different malignant cancer are still unclear. The ability of XIAP to inhibit caspases is thought to contribute chemoresistance in breast cancer and also act as adverse prognostic factor. Targeting XIAP through chemotherapeutic drugs modulates its expression and induce apoptotic threshold with the considerable growth reduction in tumor both *in vitro* and *in vivo*. Constitutive expression of XIAP results in binding and inhibition of terminal effectors in apoptotic cascade, and regulates cell survival through PI3-K/Akt pathway thereby inducing activation of Akt by phosphorylation at ser473 in the C-terminal activation domain [43]. Reciprocally, phosphorylation of XIAP by Akt has been shown to protect XIAP from ubiquitination and degradation in cancer cells [44]. Recent studies suggested that there is an intricate, coordinated regulatory system at play between XIAP and the Akt signaling pathway through feedback mechanism. The high levels of p-Akt induce activation of survival and anti-apoptotic proteins, thus reflect in cell proliferation and growth. The knocking down of XIAP is associated with activation of caspase-9 through Akt signal transduction in breast cancer cells [45]. Consequently, activated caspase-9 elevates intracellular PARP cleavage and also increase Bax/Bcl-2 ratio thus encouraging mitochondrial release of cytochrome C, resulting in apoptosis.

Utilizing the information that there is a strong interaction between XIAP and Akt in cancer, we were able to induce a synergistically potent apoptotic response after coadministration of TQ and TAM on MCF-7 and MDA-MB-231 cells. Our western blot results confirmed the antiproliferative and apoptotic role of TQ in modulating TAM’s ability to decrease cell viability. TQ and TAM in combination induced apoptosis by increasing pro-apoptotic Bax, AIF, cytochrome C and p27, and inhibiting anti-apoptotic Bcl-2 and Bcl-xl (Fig. 3A), thus shifting the balance from survival to apoptosis. TQ-TAM downregulates anti-apoptotic XIAP, resulting decrease in p-Akt levels thereby inducing procaspase-9 cleavage. Cleaved procaspase-9 activates mitochondrial release of cytochrome C by inhibiting bax/Bcl-2 ratio. Caspase-9 further induces cleavage of other procaspases as a result elevates intracellular PARP cleavage, which was further confirmed through transfection studies.

PI3K/Akt signal transduction plays a critical role in the control of cell growth and proliferation [46]. The increased Akt activation or dysregulation due to elevated Akt expression and indirect changes in Akt regulators results in enhanced cell survival signaling, which is a common feature in various forms of human cancers, including human breast carcinomas [47]. PI3K-activated (phosphorylated) Akt promotes cell survival by inhibiting apoptosis through its ability to phosphorylate/inactivate downstream targets of the apoptotic machinery, such as the pro-apoptotic Bcl-2 family member Bad and GSK-3β. These substrates directly or indirectly regulate apoptosis. GSK-3β, for example, is phosphorylated by Akt, and GSK-3β itself is involved in the regulation of cell proliferation, anti-apoptotic pathways, and cell cycle progression [48], [49]. Bad, a pro-apoptotic Bcl-2 family member is an Akt target directly implicated in regulating cell survival [47], [50]. Phosphorylation of Bad changes its affinity to Bcl-2 molecules and p-Bad is unable to inhibit Bcl-2 function [51]. We also observed downregulation of p-GSK-3β, and p-Bad by TQ-TAM treatment (Fig. 3B). At present, it is difficult to attribute the impact and individual contribution of these regulatory factors to TQ-TAM-induced apoptosis. However, it is certain that modulation of Akt activation by the combination treatment represents a major intracellular switch to mechanistically control TQ-TAM-induced tumor cell apoptosis. However, GSK-3β is not the only PI3K/Akt downstream factor potentially involved in regulating TAM-induced apoptosis. There are other downstream components of PI3K/Akt pathway that could also participate and account for the observed apoptotic effect. The expression level of MAPK increases markedly in breast cancer tissue in comparison to normal tissue and strongly correlates with axillary lymph node metastasis. Moreover, *in vitro* activated p-ERK/MAPK is expressed in cells with high metastatic potential in comparison to non-metastatic MCF-7 cells. The MAPK inhibitor CI-1041 decrease phosphorylated MAPK and reduces cell proliferation of follicular thyroid cancer and breast cancer cells *in vitro* and *in vivo* [52]. Our results also confirm that TQ-TAM in combination can be used to inhibit the MAPK/ERK pathway responsible for human breast cancer tumorigenesis and progression (Fig. 3B).

Moreover, the possible synergistic effect of TQ and TAM in inhibiting XIAP mediated Akt regulation was elucidated by overexpression of XIAP through transfection studies. To verify the role of XIAP in regulating Akt phosphorylation MCF-7 and MDA-MB-231 cells were transfected with pcDNA3-XIAP-Myc. Besides XIAP overexpression, the coadministration of TQ and TAM induce significant reduction in XIAP and p-Akt levels (Fig. 4A). This result is further supported through PARP cleavage, Caspase-9 activation and apoptotic profiling through annexin studies.

To explore the potency of TQ-TAM we had chosen siRNA-XIAP as positive control through cotransfection studies. Cotransfection studies revealed the potency of TQ-TAM in determining the extent of XIAP inhibition. In validation, we came across 3 fold decrease in MCF-7 and MDA-MB-231 cells on TQ-TAM treatment in comparison to siRNA XIAP treated control (Fig. 4D). The fold decrease of XIAP in TQ-TAM showed comparable results as that of control, thus suggesting potential of TQ-TAM in inhibiting XIAP by regulating Akt phosphorylation in inducing apoptosis.

Taken together, these results suggest synergistic inhibition of XIAP mediated p-Akt, which in turn regulates downstream targets in inducing breast cancer apoptosis. In a line, our preclinical studies showed effective approach with promising results in this synergistic combination of TQ-TAM, possibly direct future clinical development of XIAP mediated p-Akt inhibition in breast cancer.

## Supporting Information

Figure S1 **TQ enhances antiproliferative and cytotoxic effects of TAM.** Photomicrograph of MCF-7 and MDA-MB-231 cells treated with the indicated compound(s) for 24 h: (A) Cell death assessed by live/dead assay staining with Calcein AM (live, green) and Ethidium homodimer-1 (dead, red) after 24 h treatment. (B) Cell counts by Trypan blue dye exclusion assay and (C) Fluorescent micrographs of DAPI stained cells. Bars, 10 µm. The arrow indicates the nuclear blebbing in apoptotic cells. Each individual experiment has been repeated three times.
(TIF)

Figure S2 **TQ enhances anti-migratory and apoptotic activity of TAM in MCF-7 and MDA-MB-231 cells.** (A) Representative H & E stained cell images migrating into the wounded area in an *in vitro* wound healing assay at time 0 and 48 h. Bars,100 µm. (B) Bars, S.E., three random widths along the wound before and 48 h post-treatment. P<0.05. Bars, represent level of significance with P<0.05 (n = 3) with respect to control. Each individual experiment has been repeated three times.
(TIF)

Data S1 **Supplementary Methods and Results.**
(DOC)

## References

[1] Fisher B, Costantino JP (2006) Re: Tamoxifen for the prevention of breast cancer: Current status of the National Surgical Adjuvant Breast and Bowel Project P-1 study - Response. Journal of the National Cancer Institute 98: 643–644. 16670391 10.1093/jnci/djj167

[2] Jordan VC (1992) The Strategic Use of Antiestrogens to Control the Development and Growth of Breast-Cancer. Cancer 70: 977–982. 1638467

[3] Salami S, Karami-Tehrani F (2003) Biochemical studies of apoptosis induced by tamoxifen in estrogen receptor positive and negative breast cancer cell lines. Clinical Biochemistry 36: 247–253. 12810152 10.1016/s0009-9120(03)00007-9

[4] Perry RR, Kang Y, Greaves B (1995) Effects of Tamoxifen on Growth and Apoptosis of Estrogen-Dependent and Estrogen-Independent Human Breast-Cancer Cells. Annals of Surgical Oncology 2: 238–245. 7641021 10.1007/BF02307030

[5] Weng SC, Kashida Y, Kulp SK, Wang D, Brueggemeier RW, et al (2008) Sensitizing estrogen receptor-negative breast cancer cells to tamoxifen with OSU-03012, a novel celecoxib-derived phosphoinositide-dependent protein kinase-1/Akt signaling inhibitor. Mol Cancer Ther 7: 800–808. 18413793 10.1158/1535-7163.MCT-07-0434

[6] Hawkins RA, Arends MJ, Ritchie AA, Langdon S, Miller WR (2000) Tamoxifen increases apoptosis but does not influence markers of proliferation in an MCF-7 xenograft model of breast cancer. Breast 9: 96–106. 14731708 10.1054/brst.2000.0140

[7] Mandlekar S, Kong ANT (2001) Mechanisms of tamoxifen-induced apoptosis. Apoptosis 6: 469–477. 11595837 10.1023/a:1012437607881

[8] Haran EF, Maretzek AF, Goldberg I, Horowitz A, Degani H (1994) Tamoxifen enhances cell death in implanted MCF7 breast cancer by inhibiting endothelium growth. Cancer Res 54: 5511–5514. 7923186

[9] Becker M, Sommer A, Kratzschmar JR, Seidel H, Pohlenz HD, et al (2005) Distinct gene expression patterns in a tamoxifen-sensitive human mammary carcinoma xenograft and its tamoxifen-resistant subline MaCa 3366/TAM. Molecular Cancer Therapeutics 4: 151–168. 15657362

[10] Lupu R, Cardillo M, Cho C, Harris L, Hijazi M, et al (1996) The significance of heregulin in breast cancer tumor progression and drug resistance. Breast Cancer Res Treat 38: 57–66. 8825123 10.1007/BF01803784

[11] Kato S, Endoh H, Masuhiro Y, Kitamoto T, Uchiyama S, et al (1995) Activation of the Estrogen-Receptor through Phosphorylation by Mitogen-Activated Protein-Kinase. Science 270: 1491–1494. 7491495 10.1126/science.270.5241.1491

[12] Vivanco I, Sawyers CL (2002) The phosphatidylinositol 3-Kinase AKT pathway in human cancer. Nat Rev Cancer 2: 489–501. 12094235 10.1038/nrc839

[13] Perez-Tenorio G, Stal O (2002) Group SSBC (2002) Activation of AKT/PKB in breast cancer predicts a worse outcome among endocrine treated patients. British Journal of Cancer 86: 540–545. 11870534 10.1038/sj.bjc.6600126 PMC2375266

[14] Li C, Zhou C, Wang S, Feng Y, Lin W, et al (2011) Sensitization of glioma cells to tamoxifen-induced apoptosis by Pl3-kinase inhibitor through the GSK-3beta/beta-catenin signaling pathway. PLoS One 6: e27053. 22046442 10.1371/journal.pone.0027053 PMC3203172

[15] Koren J 3rd, Miyata Y, Kiray J, O'Leary JC 3rd, Nguyen L, et al (2012) Rhodacyanine derivative selectively targets cancer cells and overcomes tamoxifen resistance. PLoS One 7: e35566. 22563386 10.1371/journal.pone.0035566 PMC3338522

[16] Mimeault M, Johansson SL, Vankatraman G, Moore E, Henichart JP, et al (2007) Combined targeting of epidermal growth factor receptor and hedgehog signaling by gefitinib and cyclopamine cooperatively improves the cytotoxic effects of docetaxel on metastatic prostate cancer cells. Mol Cancer Ther 6: 967–978. 17363490 10.1158/1535-7163.MCT-06-0648

[17] Shoieb AM, Elgayyar M, Dudrick PS, Bell JL, Tithof PK (2003) In vitro inhibition of growth and induction of apoptosis in cancer cell lines by thymoquinone. International Journal of Oncology 22: 107–113. 12469192

[18] Aberg UW, Saarinen N, Abrahamsson A, Nurmi T, Engblom S, et al (2011) Tamoxifen and flaxseed alter angiogenesis regulators in normal human breast tissue in vivo. PLoS One 6: e25720. 21984941 10.1371/journal.pone.0025720 PMC3184168

[19] Rajput S, Mandal M (2012) Antitumor promoting potential of selected phytochemicals derived from spices: a review. Eur J Cancer Prev 21: 205–215. 21876437 10.1097/CEJ.0b013e32834a7f0c

[20] Hosseinzadeh H, Parvardeh S (2004) Anticonvulsant effects of thymoquinone, the major constituent of Nigella sativa seeds, in mice. Phytomedicine 11: 56–64. 14971722 10.1078/0944-7113-00376

[21] Gurung RL, Lim SN, Khaw AK, Soon JFF, Shenoy K, et al.. (2010) Thymoquinone Induces Telomere Shortening, DNA Damage and Apoptosis in Human Glioblastoma Cells. Plos One 5. 10.1371/journal.pone.0012124 PMC2920825 20711342

[22] Das S, Dey KK, Dey G, Pal I, Majumder A, et al (2012) Antineoplastic and apoptotic potential of traditional medicines thymoquinone and diosgenin in squamous cell carcinoma. PLoS One 7: e46641. 23077516 10.1371/journal.pone.0046641 PMC3471895

[23] Gali-Muhtasib H, Roessner A, Schneider-Stock R (2006) Thymoquinone: A promising anti-cancer drug from natural sources. International Journal of Biochemistry & Cell Biology 38: 1249–1253. 16314136 10.1016/j.biocel.2005.10.009

[24] Somanath PR, Razorenova OV, Chen JH, Byzova TV (2006) Akt1 in endothelial cell and angiogenesis. Cell Cycle 5: 512–518. 16552185 10.4161/cc.5.5.2538 PMC1569947

[25] Murphy DA, Makonnen S, Lassoued W, Feldman MD, Carter C, et al (2006) Inhibition of tumor endothelial ERK activation, angiogenesis, and tumor growth by sorafenib (BAY43–9006). American Journal of Pathology 169: 1875–1885. 17071608 10.2353/ajpath.2006.050711 PMC1780219

[26] Sethi G, Ahn KS, Aggarwal BB (2008) Targeting nuclear factor-kappa B activation pathway by thymoquinone: role in suppression of antiapoptotic gene products and enhancement of apoptosis. Mol Cancer Res 6: 1059–1070. 18567808 10.1158/1541-7786.MCR-07-2088

[27] Yi T, Cho SG, Yi Z, Pang X, Rodriguez M, et al (2008) Thymoquinone inhibits tumor angiogenesis and tumor growth through suppressing AKT and extracellular signal-regulated kinase signaling pathways. Mol Cancer Ther 7: 1789–1796. 18644991 10.1158/1535-7163.MCT-08-0124 PMC2587125

[28] Younes MN, Yigitbasi OG, Park YW, Kim SJ, Jasser SA, et al (2005) Antivascular therapy of human follicular thyroid cancer experimental bone metastasis by blockade of epidermal growth factor receptor and vascular growth factor receptor phosphorylation. Cancer Research 65: 4716–4727. 15930290 10.1158/0008-5472.CAN-04-4196

[29] Dash R, Mandal M, Ghosh SK, Kundu SC (2008) Silk sericin protein of tropical tasar silkworm inhibits UVB-induced apoptosis in human skin keratinocytes. Molecular and Cellular Biochemistry 311: 111–119. 18214642 10.1007/s11010-008-9702-z

[30] Sarkar S, Mazumdar A, Dash R, Sarkar D, Fisher PB, et al (2011) ZD6474 Enhances Paclitaxel Antiproliferative and Apoptotic Effects in Breast Carcinoma Cells. Journal of Cellular Physiology 226: 375–384. 20665703 10.1002/jcp.22343

[31] Mandal M, Adam L, Kumar R (1999) Redistribution of activated caspase-3 to the nucleus during butyric acid-induced apoptosis. Biochem Biophys Res Commun 260: 775–780. 10403841 10.1006/bbrc.1999.0966

[32] Sarkar S, Mazumdar A, Dash R, Sarkar D, Fisher PB, et al (2010) ZD6474, a dual tyrosine kinase inhibitor of EGFR and VEGFR-2, inhibits MAPK/ERK and AKT/PI3-K and induces apoptosis in breast cancer cells. Cancer Biology & Therapy 9: 592–603. 20139705 10.4161/cbt.9.8.11103

[33] Venkatesan P, Puvvada N, Dash R, Kumar BNP, Sarkar D, et al (2011) The potential of celecoxib-loaded hydroxyapatite-chitosan nanocomposite for the treatment of colon cancer. Biomaterials 32: 3794–3806. 21392822 10.1016/j.biomaterials.2011.01.027

[34] Emdad L, Lee SG, Su ZZ, Jeon HY, Boukerche H, et al (2009) Astrocyte elevated gene-1 (AEG-1) functions as an oncogene and regulates angiogenesis. Proc Natl Acad Sci U S A 106: 21300–21305. 19940250 10.1073/pnas.0910936106 PMC2795510

[35] Barnes CJ, Bagheri-Yarmand R, Mandal M, Yang ZB, Clayman GL, et al (2003) Suppression of epidermal growth factor receptor, mitogen-activated protein kinase, and Pak1 pathways and invasiveness of human cutaneous squamous cancer cells by the tyrosine kinase inhibitor ZD1839 (Iressa). Molecular Cancer Therapeutics 2: 345–351. 12700278

[36] Santhekadur PK, Gredler R, Chen D, Siddiq A, Shen XN, et al.. (2011) LSF (Late SV40 Factor) enhances angiogenesis by transcriptionally upregulating matrix metalloproteinase-9 (MMP-9). J Biol Chem. 10.1074/jbc.M111.298976 PMC3270996 22167195

[37] Mandal M, Myers JN, Lippman SM, Johnson FM, Williams MD, et al (2008) Epithelial to mesenchymal transition in head and neck squamous carcinoma - Association of src activation with E-cadherin down-regulation, vimentin expression, and aggressive tumor features. Cancer 112: 2088–2100. 18327819 10.1002/cncr.23410

[38] Sarkar D, Park ES, Emdad L, Lee SG, Su ZZ, et al (2008) Molecular basis of nuclear factor-kappaB activation by astrocyte elevated gene-1. Cancer Res 68: 1478–1484. 18316612 10.1158/0008-5472.CAN-07-6164

[39] Jordan NJ, Gee JMW, Barrow D, Wakeling AE, Nicholson RI (2004) Increased constitutive activity of PKB/Akt in tamoxifen resistant breast cancer MCF-7 cells. Breast Cancer Research and Treatment 87: 167–180. 15377841 10.1023/B:BREA.0000041623.21338.47

[40] Asselin E, Mills GB, Tsang BK (2001) XIAP regulates Akt activity and caspase-3-dependent cleavage during cisplatin-induced apoptosis in human ovarian epithelial cancer cells. Cancer Res 61: 1862–1868. 11280739

[41] Dorai T, Aggarwal BB (2004) Role of chemopreventive agents in cancer therapy. Cancer Letters 215: 129–140. 15488631 10.1016/j.canlet.2004.07.013

[42] Gali-Muhtasib H, Diab-Assaf M, Boltze C, Al-Hmaira J, Hartig R, et al (2004) Thymoquinone extracted from black seed triggers apoptotic cell death in human colorectal cancer cells via a p53-dependent mechanism. International Journal of Oncology 25: 857–866. 15375533

[43] Hussain AR, Uddin S, Ahmed M, Bu R, Ahmed SO, et al (2010) Prognostic significance of XIAP expression in DLBCL and effect of its inhibition on AKT signalling. J Pathol 222: 180–190. 20632385 10.1002/path.2747

[44] Gagnon V, Van Themsche C, Turner S, Leblanc V, Asselin E (2008) Akt and XIAP regulate the sensitivity of human uterine cancer cells to cisplatin, doxorubicin and taxol. Apoptosis 13: 259–271. 18071906 10.1007/s10495-007-0165-6

[45] Aird KM, Ding X, Baras A, Wei J, Morse MA, et al (2008) Trastuzumab signaling in ErbB2-overexpressing inflammatory breast cancer correlates with X-linked inhibitor of apoptosis protein expression. Mol Cancer Ther 7: 38–47. 18202008 10.1158/1535-7163.MCT-07-0370

[46] Franke TF, Hornik CP, Segev L, Shostak GA, Sugimoto C (2003) PI3K/Akt and apoptosis: size matters. Oncogene 22: 8983–8998. 14663477 10.1038/sj.onc.1207115

[47] Hlobilkova A, Knillova J, Svachova M, Skypalova P, Krystof V, et al (2006) Tumour suppressor PTEN regulates cell cycle and protein kinase B/Akt pathway in breast cancer cells. Anticancer Research 26: 1015–1022. 16619501

[48] Franke TF, Cantley LC (1997) Apoptosis - A Bad kinase makes good. Nature 390: 116–117. 9367147 10.1038/36442

[49] Pap M, Cooper GM (1998) Role of glycogen synthase kinase-3 in the phosphatidylinositol 3-kinase/Akt cell survival pathway. Journal of Biological Chemistry 273: 19929–19932. 9685326 10.1074/jbc.273.32.19929

[50] Ren Y, Wu H (2003) Simultaneous suppression of erk and Akt/PKB activation by a Gab1 pleckstrin homology (PH) domain decoy. Anticancer Research 23: 3231–3236. 12926057

[51] Liu Y, Sun SY, Owonikoko TK, Sica GL, Curran WJ, et al (2012) Rapamycin induces Bad phosphorylation in association with its resistance to human lung cancer cells. Mol Cancer Ther 11: 45–56. 22057915 10.1158/1535-7163.MCT-11-0578 PMC3256262

[52] Reddy KB, Glaros S (2007) Inhibition of the MAP kinase activity suppresses estrogen-induced breast tumor growth both in vitro and in vivo. International Journal of Oncology 30: 971–975. 17332937

