# Supplementary figures and images for: Targeted Apoptotic Effects of Thymoquinone and Tamoxifen on XIAP Mediated Akt Regulation in Breast Cancer
Source: PLoS One. 2013 Apr 17;8(4):e61342. doi: 10.1371/journal.pone.0061342 (PMC3629226; doi:10.1371/journal.pone.0061342)

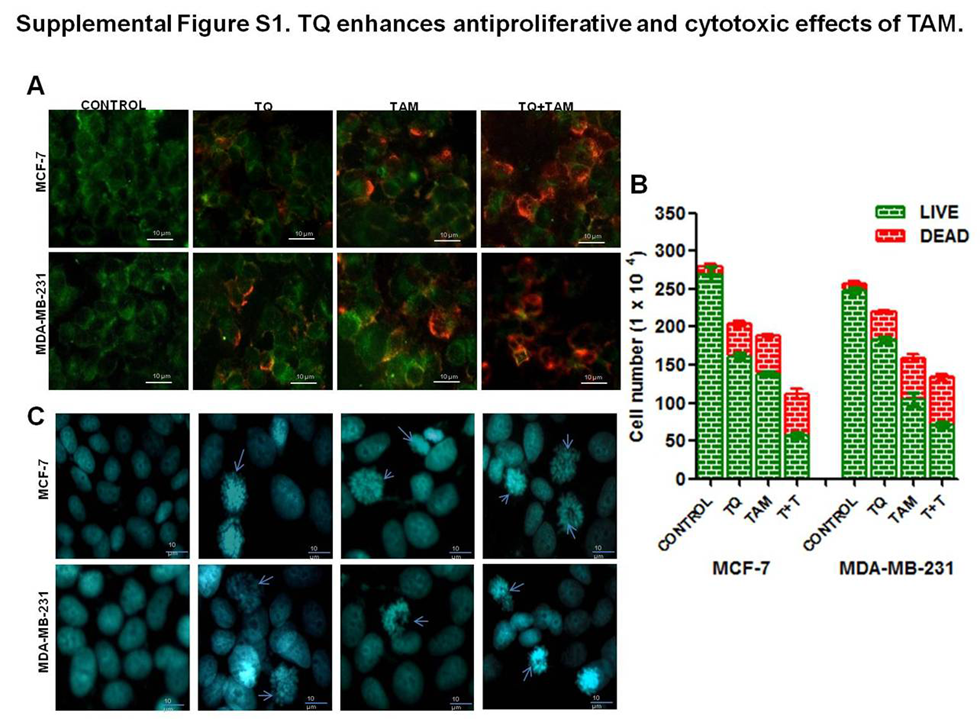

Supplement: Figure S1 — TQ enhances antiproliferative and cytotoxic effects of TAM. Photomicrograph of MCF-7 and MDA-MB-231 cells treated with the indicated compound(s) for 24 h: (A) Cell death assessed by live/dead assay staining with Calcein AM (live, green) and Ethidium homodimer-1 (dead, red) after 24 h treatment. (B) Cell counts by Trypan blue dye exclusion assay and (C) Fluorescent micrographs of DAPI stained cells. Bars, 10 µm. The arrow indicates the nuclear blebbing in apoptotic cells. Each individual experiment has been repeated three times. (TIF) [file pone.0061342.s001.tif]

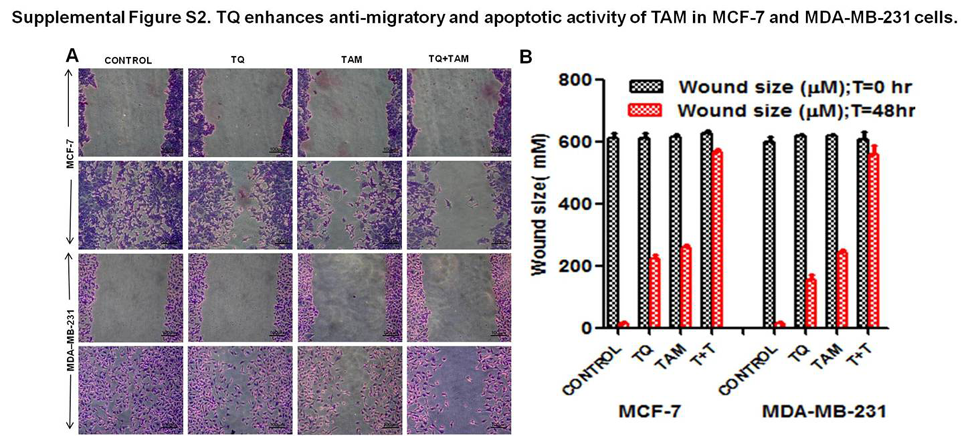

Supplement: Figure S2 — TQ enhances anti-migratory and apoptotic activity of TAM in MCF-7 and MDA-MB-231 cells. (A) Representative H & E stained cell images migrating into the wounded area in an in vitro wound healing assay at time 0 and 48 h. Bars,100 µm. (B) Bars, S.E., three random widths along the wound before and 48 h post-treatment. P<0.05. Bars, represent level of significance with P<0.05 (n = 3) with respect to control. Each individual experiment has been repeated three times. (TIF) [file pone.0061342.s002.tif]
