# Supplementary material for: Targeted Apoptotic Effects of Thymoquinone and Tamoxifen on XIAP Mediated Akt Regulation in Breast Cancer
Source: PLoS One. 2013 Apr 17;8(4):e61342. doi: 10.1371/journal.pone.0061342 (PMC3629226; doi:10.1371/journal.pone.0061342)
Supplement: Data S1 — Supplementary Methods and Results. (DOC) [file pone.0061342.s003.doc]

**Title: Targeted apoptotic effects of thymoquinone and tamoxifen on XIAP mediated Akt regulation in breast cancer**

**Supplemental Methods and Results**

**Morphological studies**

MCF-7 and MDA-MB-231 cells were plated on coverslips in DMEM complete medium. After 1 d, cells were treated with IC_50_ values of TQ and/or TAM for 24 h. After incubation, cells were examined using phase contrast microscopy. Cells were fixed using 3.7% paraformaldehyde and permeabilized with 0.1 % Triton X-100 and stained with DAPI as per manufacturer’s instructions. Cell viability was assessed by cell staining with Calcein AM and Ethidium homodimer-1 using the LIVE/DEAD**®** Viability/Cytotoxicity kit as per the manufacturer’s instructions and trypan blue dye exclusion assay. Cells were analyzed by confocal laser scanning microscopy (Olympus FluoView FV1000, Version 1.7.1.0, TYO, Japan) using the appropriate wavelength. Images were captured and digitized using FLUOVIEW 1000 (Version 1.2.4.0) imaging software, TYO, Japan.

**Chick Chorioallantoic Membrane (CAM) assay**

To determine *in vivo* antiangiogenic activity of TQ and/or TAM, CAM and HUVEC tube formation assays were performed as described previously with some modifications. Two day-old fertilized eggs were incubated at 37°C in 60-70% relative humidity. After 5 d of incubation, a 1-2 cm^2^ window was opened and a sterile round filter paper (5-mm in diameter, Whatman qualitative filter papers, Sigma-Aldrich, St. Louis, MO, USA) containing serum-free medium (SFM) alone as control, VEGF or TQ and/or TAM (IC_50_ concentrations/filter paper) was applied onto the CAM of individual embryos. After 2 d incubation, the upper eggshell was removed and capillaries within 2.5 mm around the filter paper were observed and photographed under a stereomicroscope (Olympus, SZX16, PA, USA).

**Capillary-Like Tube Formation (HUVEC) assay**

For tube formation assays, growth factor-depleted Matrigel was applied to a 96-well tissue culture plate (50 μl per well). After polymerization of the Matrigel (37°C, 1 h), 2 h serum starved HUVECs were harvested by using trypsin/EDTA, washed with assay medium, and seeded at a density of 7.5 × 10^3^ cells/well (final volume, 50 μl) on the polymerized Matrigel in the presence or absence of 30 ng/ml VEGF along with TQ and/or TAM treatment. The plate was incubated at 37°C for 24 h, and then the medium was aspirated and cells were fixed in neutral buffered 10% formalin. Representative pictures were taken at 10× magnification.

**Boyden chamber assay**

To test the anti-invasive effect of TAM and/or TQ, 8-µm filters were coated with Matrigel (20 µg/ filter) and placed in Boyden chambers. MDA-MB-231 Cells (1 × 10^5^), suspended in DMEM containing 0.1% BSA and treated with TQ and/or TAM, were added to the top chamber. Conditioned medium from mouse fibroblast NIH/3T3 cells was used as a source of chemoattractant and placed in the bottom compartment of the Boyden chamber. After 24 h incubation at 37°C, non-invading cells were scraped off, and cells that had migrated to the lower surface of the filter inserts were fixed with 100% methanol for 10 min and stained with hematoxylin- and eosin (H&E). The results are expressed as the percent of migrated cells as compared to the control (untreated cells). Each experiment was performed three times with triplicate samples.

***In vitro* wound healing (scratch) assay**

To test the effect of TAM and/or TQ on cell migration, 1 × 10^5^ cells of MCF-7 and MDA-MB-231 were plated in 6-well tissue culture plates and grown for 24 h to obtain a confluent monolayer and migration was studied by *in vitro* wounding (scratch) assay with slight modifications. The monolayer was scraped (wounded) in a straight line to create a ‘‘wound’’ with a 200-μl pipette tip. The debris was removed and the edge of the wound was made smooth by washing the cells once with 1 ml of the growth medium and then replaced with 3 ml of complete media along with TQ and/or TAM. Cells were observed 48 h post-treatment. Cells invading the wound (scratch) line were observed under an inverted phase-contrast microscope after H&E staining. Cells invading the wound line were observed under an inverted phase-contrast microscope using 10×, Leica DMR, Germany. The distance between the two sides of the scratch was measured after the indicated time intervals using the Leica QWin software, IL, USA. Each experiment was performed three times with triplicate samples.

**RESULTS**

**TQ inhibits cell proliferation and induces apoptosis in combination with TAM**

Cell viability is a dynamic process that reflects a balance between cell proliferation and cell death. To define the contributory roles of proliferation and apoptosis, and nuclear morphological changes, trypan blue dye exclusion tests (Fig. S1B) and nucleus staining with fluorescent dye (DAPI) (Fig. S1C), respectively, were performed. Decreased cell viability was a consequence of both the growth inhibitory and apoptotic effects of TQ when combined with TAM. There was >50% cell death in combinatorial treated cells as compared to control cells (Fig. S1B). LIVE/DEAD cytotoxicity assays (Fig. S1A) confirmed the increased percentage of apoptotic cells in TQ or TAM treated group in comparison to control, which was further enhanced following combination treatment.

**TQ augments TAM’s efficacy in inhibiting *in vitro* cell migration**

To determine the effect of TQ and/or TAM on migration, *in vitro* wound (scratch) assays were performed in both MCF-7 and MDA-MB-231 cells. The size of the wound (scratch) before treatment was 558.00 ± 9.54 (mean ± S.E.), which was decreased to 46.00 ± 18.21 (control), 199.00 ± 16.21 (TQ), 266.00 ± 26.58 (TAM) and 489.79 ± 22.73 (TQ plus TAM) in MCF-7 cells. In the case of MDA-MB-231, wound size (scratch) prior to treatment was 602.00 ± 7.27, which was decreased to 39.73 ± 17.56 (control), 198.11 ± 17.56 (TQ), 260.3 ± 42.67 (TAM) and 489.7 ± 25.58 (TQ-TAM) treatment 48 h post-treatment (Fig. S2A and S2B). These results confirm that TQ in combination with TAM effectively blocks cell migration of MCF-7 and MDA-MB-231 cells (Fig. S2B) and inhibited wound healing, as there was no significant change in wound size of both MCF-7 and MDA-MB-231 cells 48 h post-treatment with the combination of TQ and TAM as compared to the initial time of treatment.
